# Cochlear implant-specific risks should be considered, when assessing the quality of life of children and adolescents with hearing loss and cochlear implants–not just cochlear implant-specific benefits–Perspective

**DOI:** 10.3389/fnins.2022.985230

**Published:** 2022-11-08

**Authors:** Maria Huber

**Affiliations:** Department of Otorhinolaryngology, Head and Neck Surgery, Paracelsus Medical University, Salzburg, Austria

**Keywords:** hearing loss, children and adolescents, cochlear implants, quality of life, CI-specific risks

## Abstract

Cochlear implants (CIs) are electronic medical devices that enable hearing in cases where traditional hearing aids are of minimal or no use. Quality of life (QoL) studies of children and adolescents with a CI have so far focused on the CI-specific benefits. However, the CI-specific risks listed by the U.S. Food and Drug Administration have not yet been considered. From this list, medical and device-related complications, lifelong dependency on the implanted device, and neurosecurity risks (CI technology is an interface technology) may be particularly relevant for young CI users. Medical and device-related complications can cause physical discomfort (e.g., fever, pain), as well as functioning problems (e.g., in speech discrimination, social behavior, and mood). In the worst case, reimplantation is required. Clinical experience shows that these complications are perceived as a burden for young CI users. Furthermore, many young patients are worried about possible complications. Additionally, CIs can be at least a temporary burden when children, typically at the age of 8–9 years, realize that they need the CI for life, or when they become peer victims because of their CI. Concerning neurosecurity risks, it is still unknown how young CI recipients perceive them. In summary, CI-specific risks can be perceived as a burden by young CI users that impairs their QoL. Therefore, they should not be ignored. There is an urgent need for studies on this topic, which would not only be important for professionals and parents, but also for the design of CI-specific QoL instruments.

## Introduction

**FIGURE 1:**
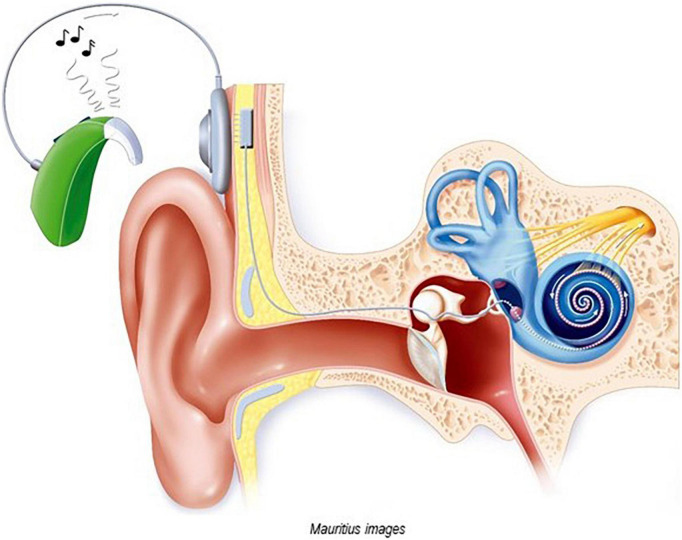
Image of a cochlear implant (with permission of Mauritius Images, Kd 55911). The external sound processor converts sound into a sequence of electrical impulses and sends these signals *via* a transmitter coil to the internal receiver-stimulator. The internal device, which is located under the skin in a cavity created in the skull bone, processes the electrical signal and transmits them *via* electrodes to the spiral ganglion cells of the cochlear nerve. Both internal and external devices are equipped with magnets.

Cochlear implants (CIs) are electronic medical devices that bypass the hair cells in the cochlea and directly stimulate the auditory nerve (Clark et al., 1977; Desoyer and Hochmair, 1977), see Figure 1. They allow people with severe and profound hearing loss “to receive and process sounds and speech” (Medlineplus),^1^ where traditional hearing aids have been of little or no use. A CI consists of an external device, the sound processor, and an internal device, the receiver-stimulator (Figure 1), as well as an electrode-to-neural interface (DiNino et al., 2019; Shader et al., 2020).

Numerous studies indicate that children and adolescents with congenital or early onset severe and profound hearing loss benefit from CI: it provides a clear benefit in the development of brain regions associated with hearing (Sharma et al., 2002; Kral and Sharma, 2012; Kral et al., 2016; Cardon and Sharma, 2019; Lee et al., 2020; Wang et al., 2021) and supports auditory development (Purcell et al., 2021) as well as the development of spoken language (Percy-Smith et al., 2008; Peters et al., 2010; Geers et al., 2016). In particular, very early implantation (Dettman et al., 2016; Ruben, 2018; Sharma et al., 2020; Naik et al., 2021) and bilateral implantations (Lieu et al., 2020; Sharma et al., 2020) benefit the development of verbal language. For example, 64% of children who were implanted before the age of 12 months showed receptive and expressive language skills in the normative range at school entry (Dettman et al., 2016). In the long term, CIs increase the chance for school and professional training in the hearing world (Huber et al., 2008, 2014; Huber and Kipman, 2012; Sarant et al., 2015).

Several studies reported positive correlations between these CI-specific benefits and the quality of life (QoL) of children and adolescents with hearing loss, such as speech recognition, especially in noisy environments, spoken language skills (Huber, 2005; Haukedal et al., 2018, 2020; Suneel et al., 2020; Ching et al., 2021), and education (Van der Straaten et al., 2020).

However, there are also CI-specific risks listed by the U.S. Food and Drug Administration (FDA^2^). From this list, the medical and device-related complications, lifelong dependency on the implanted device, and neurological safety risks may be particularly relevant for young CI users. Although these specific risks may also be related to QoL, to our knowledge they have not yet been considered in assessing the QoL of young CI recipients. Furthermore, little is known about how young recipients perceive and experience these risks.

“Quality of life” refers to different areas of a person’s life, such as economic status, rights, culture, and health (Fayed et al., 2012) with “health-related quality of life” or HRQoL being commonly regarded as a sub-domain of the more global concept of QoL (World Health Organization [Who], 1948; Davis et al., 2006). Usually, HRQoL models include physical health, mental health, and social health. According to the well-validated model of Wilson and Cleary’s (1995), Bakas et al. (2012), Ojelabi et al. (2017), HRQoL is the result of (a) biological/physiological variables, (b) symptom status, (c) functional status, and (d) subjective perception of one’s own state of health.^3^

Generic or cross-disease HRQoL tools allow for comparison between groups, e.g., between people with hearing loss and people with normal hearing. Disease-specific HRQoL instruments are necessary for the assessment of the impact of therapeutic changes (Wiebe et al., 2003). In addition, they provide information about how great the subjective burden of a specific disease is for the individual affected.

As with adults, there is no common definition of QoL for children (Drotar, 2004; Davis et al., 2006; Fayed et al., 2012; Ravens-Sieberer et al., 2014a,b; Wallander and Koot, 2016). However, there is a consensus that valid tools for the assessment of pediatric QoL have to be child-specific, as stated by the World Health Organization [Who] (1994).

A consensus exists that the children and adolescents themselves are the best informants of their own QoL (Riley, 2004; Davis et al., 2006; Upton et al., 2008; Ellert et al., 2011; Ravens-Sieberer et al., 2014a,b). Children are quite capable of reporting on their state of health as early as 5 years of age (Riley, 2004). From the age of eight, children provide reliable information about their health experiences (Rebok et al., 2001) and their attitude toward their illness, worries, and hopes. They also draw their conclusions from their parents’ behavior and perceive parental concerns (Wollenhaupt et al., 2012; Beacham and Deatrick, 2015; Blackwell et al., 2019).

Recently, the development of pediatric CI-specific QoL instruments was initiated (Hoffman et al., 2019; Cejas et al., 2021). In these instruments, some burdens were also considered (e.g., “fatigue” Hoffman et al., 2019). However, the domains and items dealing with these burdens are kept so general that they apply to all young people with hearing loss with and without a CI (e.g., Bess et al., 2016). Potential CI-specific risks (see FAD) were not specifically addressed.

## Perspective: Cochlear implant-specific risks can be a burden and should, therefore, be considered when assessing the quality of life studies of young cochlear implant recipients

Cochlear implant-specific risks could have direct or indirect, mediating, or moderating negative effects on the functioning and subjective perception of young CI users, with possible consequences for their QoL.

### Medical and device-related complications and related burdens

There is consensus that cochlear implants are largely safe across all age groups with congenital and acquired hearing loss, even for very young children (Rajan et al., 2018; Uecker et al., 2019; Sharma et al., 2020; Deep et al., 2021; Naik et al., 2021; Purcell et al., 2021). The survival rates for cochlear implants are as follows [Lane et al. (2019): 10-year cumulative survival rates 97.2%; Chen et al. (2022): 10-year rate 96.8%, 20-year rate 96.7%].

The most often observed reasons for surgery due to *medical complications*^4^ are device infection and mastoiditis [e.g., Vila et al. (2017): 3.9% for device infections, Nisenbaum et al. (2020): 3.7% for device infections and mastoiditis, Deep et al. (2021): 0.8% “concern for device infection,” 1.2% mastoiditis]. Most pediatric infections occur within 180 days after surgery (Lander et al., 2020). Less often observed reasons (reimplantation, post-implantation) are cerebrospinal fluid leakage, device migration, electrode misinsertion, electrode displacement, hematoma, and facial paralysis (Yeung et al., 2018; Chen et al., 2022). Therefore, even in later years, it cannot be ruled out that reimplantation will be necessary (Chen et al., 2022).

*Device failures* are more common than medical complications. They can occur at any time and range from 0.8 to 5.7% of hard failures and 0.8 to 8.9% of soft failures (Lane et al., 2019; Yosefof et al., 2021). A cochlear implant *hard* failure is defined as a “complete loss of connection between the external and internal device,” mainly due to damages in the internal device (Bhadania et al., 2018) and is identified with an objective test. A cochlear implant *soft* failure “…is an uncommon occurrence in which a device malfunction is suspected but cannot be proven using currently available *in vivo* methods” (Balkany et al., 2005).^5^

Hard failures require reimplantation more frequently than soft failures (Yeung et al., 2018; Chen et al., 2022). In the case of soft failures, in particular, months or years can pass between the onset of the symptoms and the replacement surgery (Yosefof et al., 2021).

In addition, there are also *electrode abnormalities* with an incidence rate of about 9% (Harris et al., 2020). According to Harris et al. (2020), faulty electrodes in children with CI were associated with social isolation and anxiety.

To my knowledge, no study has so far addressed the extent to which these CI-specific complications are related to functional problems such as fatigue, reduced language performance, stress, and anxiety in the short or long term. My clinical experience with more than 40 affected young patients with CI shows that complications are perceived as a burden.

Additionally, many young CI recipients are concerned that these complications can occur in the future. According to a previous study, one-third of young people with CIs are stressed and worried about potential device failures, despite having a clearly positive attitude toward their own cochlear implants (Wheeler et al., 2007). According to other studies, parents are also stressed and worried because of these possible complications (Archbold et al., 2002; Okubo et al., 2008; Fitzpatrick et al., 2011), which can also affect the subjective health perception of children and adolescents (Wollenhaupt et al., 2012; Beacham and Deatrick, 2015; Blackwell et al., 2019).

### Dependency on the device and related burdens

In most European countries, it is estimated that at least 65% are implanted under the age of 3.5 years and at least 50% are implanted under the age of 12 months (Lammers et al., 2015). At this age, they are not developmentally mature enough to understand their condition and the treatment. However, based on my clinical experience with more than 200 children with CI, as children get older, they may increasingly become aware of both their hearing problems and the benefits of their CI. By the age of 8–9, they are usually fully aware that the individuals in their surroundings do not have hearing problems, that the hearing condition does not heal, and that they depend on CIs while others do not.^6^ As a result, some children may experience temporary difficulties in dealing with these new insights. I have observed isolated cases (about 10 out of 200) of older children who, overnight and for no apparent reason, temporarily became very thoughtful and exhibited an ambivalent or negative attitude toward their CI. For older adults with age-related hearing loss (presbycusis), acceptance of hearing aids appears to be related to acceptance of their own hearing loss (Humes and Dubno, 2021). Despite having clear comprehension and communication problems, those who denied their hearing loss refrained from using hearing aids (Humes and Dubno, 2021). There was also a fear of being stigmatized for using hearing aids (Barker et al., 2017; Vas et al., 2017).

Assuming that even in children, an ambivalent or negative attitude toward the hearing aid (CI) can go hand in hand with a denial of hearing loss, greater pressure must be expected in children than in adults. Children with severe-profound hearing loss (that is why they need a CI) may be more dependent on their device—and may be more aware of their dependency—than the elderly with bilateral high-frequency hearing loss. In addition, they may be more dependent on their device (CI) than children with mild to moderate hearing loss (hearing aids).

Stigmatization can also occur in children and adolescents with CI. For example, children become peer victims because of their devices. In this case, dependency on the CI will be experienced as stressful. Feijóo et al. (2021) reported increased prevalence rates of peer victimization of young CI users compared to normal-hearing peers.^7^ Warner-Czyz et al. (2018) found higher rates among young CI users compared to adolescents with hearing aids. A possible reason for this may be that CIs are more visible than hearing aids (Feijóo et al., 2021). Therefore, CI dependency may temporarily lead to grief and social withdrawal in children and adolescents. However, specific studies addressing these issues are still needed.

### Neurosecurity risks and related burdens

Today’s telehealth programs allow for online reprograming of CIs.^8^ Furthermore, CIs can communicate with smartphones, iPads, and computers allowing young CI recipients and their parents some control over their audio processor settings. However, this also means the possibility and risk of unauthorized reprograming.^9^ Although unlikely in practice, CIs theoretically can be hacked (Capkun and Bodner, 2010; Tabasum et al., 2018; Hansson, 2020). CIs are neuroprosthesis. To the author’s knowledge, countermeasures to protect their “neurosecurity” (Denning et al., 2009; Burwell et al., 2017) are not published. The manufacturers’ websites contain general statements on the subject of cybersecurity, but these hardly deal with the specific problem of neurosecurity of CI.

It is still unknown, whether and to what extent older children and adolescents with CI are even aware of these risks. In my experience, hearing aid acousticians avoid addressing this topic because, according to their own statements, they do not want to unsettle the young CI recipients. However, it cannot be excluded that the patients themselves will come up with the problem and develop their own theories. Research addressing the consequences of missing information is, therefore, warranted.

## Final discussion

In summary, it cannot be ruled out that the CI-specific risks of medical and device complications (a), dependency on CIs (b), and neurosecurity issues (c) will be perceived as a burden for young CI recipients, at least for some of them, and at least temporarily. The mere possibility of device failure, peer victimization, or cybersecurity breaches may have already a negative impact on QoL.

The percentage of those who are worried about the eventuality of risk (a) with a negative impact on QoL may be higher than those who are actually affected by this complication, which may also affect QoL.

The number of young patients who find it difficult to cope with the prospect of life-long dependency [risk (b)] is not known. Furthermore, actual peer victimization but also its mere possibility and possible long-term consequences may have a negative impact on QoL. As exact numbers are not known, this topic warrants further research, e.g., studies addressing the percentage of those with coping problems, the percentage of peer victims due to CI, and the impact of these problems on QoL.

Only a few young CI users may be concerned with risk (c), possibly because their knowledge of CI technology and its potential vulnerability may be low (Wheeler et al., 2007). Overall, it can be stated that there seem to be no obvious indications of possible burdens due to risk (c). Studies on normal-hearing adolescents show that knowledge about the cybersecurity of their smartphones is low (Mai and Tick, 2021). Studies that deal with the knowledge about the cybersecurity of young CI users are still missing. In addition, surveys on the frequency of cybersecurity problems and studies that deal with possible concerns of young CI wearers are missing. Worry can affect QoL.

How are these CI-specific risks related to QoL? In the event of an actual medical or device complication (risk a) and with a generic pediatric HRQoL approach for children, all aspects of quality of life (physical health, mental health, social health, friends, school, and possibly family) may be impacted. Concerns among young CI recipients about potential complications are assumed to have the greatest impact on the mental health domain. Previous studies do not indicate increased anxiety and emotional problems in young CI recipients compared to their normal-hearing peers (Theunissen et al., 2012; Huber et al., 2015). However, studies looking at long-term mental health effects in young CI users who actually experienced these complications are still lacking. In addition, to my knowledge, there are no studies that take into account the variables “actual experience of complications” and “concerns about the eventuality.” Further studies are needed to address the impact of actual complications on academic skills.

Furthermore, in the case of risk (b), the domains of mental health, social health, friends, school, and family are possibly affected. Especially in the case of peer victimization, the mental health domain may be impacted. Peer victimization of normal-hearing children and adolescents was found to be positively correlated to social anxiety (Pontillo et al., 2019). Furthermore, stress reactions (skin conductance level, heart rate, and affective reactions) of young normal-hearing adults as a consequence of social exclusion were found to depend more on previous experiences of peer victimization and less on the diagnosis of social anxiety disorder of these individuals (Iffland et al., 2014). However, no studies have been conducted with young CI users to address these issues, such as studies on the possible long-term mental health effects of peer victimization.

In risk (c), we cannot exclude, that the mental health domain and the social domains are affected.

All risks can be directly and indirectly (*via* the parents) related to the functioning and self-perception of the young CI recipients.

Overall, if the magnitude of the burden is defined as the sum of all impairments in quality of life, due to risks a–c, the burden may be higher for older children and adolescents, than for younger children. The latter still have little insight into their condition and CI technology and are too young to be able to ask questions about cybersecurity and neurosafety.

Regarding regional differences, I do not expect differences for (a) and (b). For (c), a rating is not possible for me.

**FIGURE 2:**
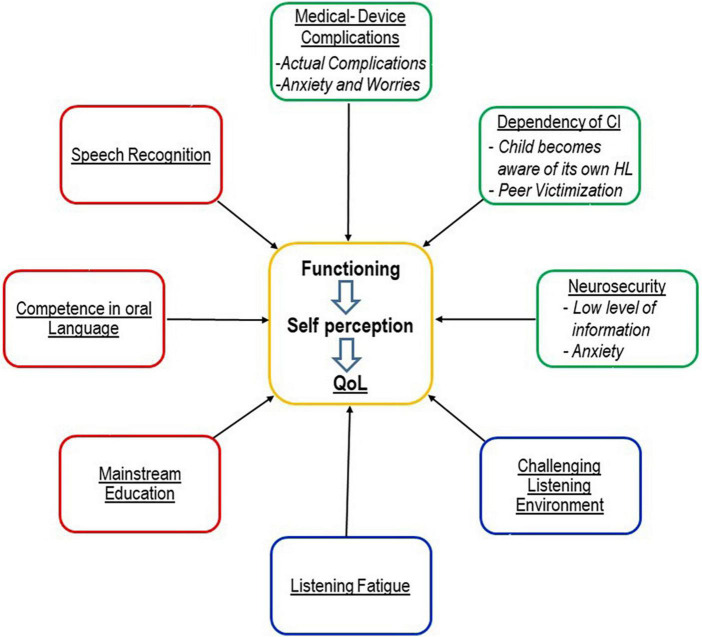
Model illustrating CI-specific advantages and risks as possible influencing variables on QoL [following the model of Wilson and Cleary (1995)] of older children and adolescents with CI. Please note that this is a simplified illustration since possible associations and interactions between the variables are not shown and there is the assumption that the impact of variables can gain and lose weight over time. This possible dynamic was not illustrated in the graph; and individual characteristics, as well as socioeconomic and educational background, have also an impact on QoL but are not shown in the graph. Red: CI-specific benefit. Blue: Burden specific for hearing loss (Umansky et al., 2011; Rachakonda et al., 2014). Green: CI-specific risk.

Studies on this topic are still largely lacking. There is a need for observational studies and case series. The results of these studies would also be important for the design of CI-specific pediatric QoL instruments, see Figure 2. The results of these studies would be relevant for the clinical work of surgeons, audiologists, therapists, teachers, and parents.

Furthermore, because children also have a right to information (De Lourdes Levy et al., 2003), they should be informed about medical and device-related complications, as well as cybersecurity and neurosecurity issues.

Finally, it would be important for manufacturers to update and supplement information on CI technology, cybersecurity, and neurosecurity on their websites. This information should also be provided in child-friendly language.

For the first time, this perspective article addressed possible problem areas for QoL that have so far largely gone unnoticed. This is a strength of this perspective article. However, studies addressing these issues are still lacking. Some of the findings presented herein are the experiences of the author with her patients. However, these are recurrent issues, which have motivated this perspective. The author believes that controlled studies are warranted to address the questions raised herein. Furthermore, the situation of children with additional special needs and CI was not taken into account in this article.

## Conclusion

In summary, while CIs have enormous benefits, the specific risks and possible consequences of these risks should be taken into account when evaluating QoL. There is an urgent need for studies addressing this issue.

## Data availability statement

The original contributions presented in this study are included in the article/supplementary material, further inquiries can be directed to the corresponding author.

## Author contributions

MH agreed to be accountable for the content of the work and approved the submitted version.

## Footnotes

1 National Library of Medicine https://medlineplus.gov/ency/article/007203.htm.

2 FDA https://www.fda.gov/medical-devices/cochlear-implants/benefits-and-risks-cochlear-implants.

3 Although the HRQoL approach might be the most commonly used in the healthcare sector, there are other QoL approaches, e.g., the subjective wellbeing (SWB) concept, see the reviews in Ravens-Sieberer et al. (2014a) and Wallander and Koot (2016).

4 According to the FDA, risks arise from the “surgical implant procedure” and patients may “… have their implant fail.” or “have to have it removed temporarily or permanently if an infection develops.”

5 To identify soft failures, “detectable hardware- or software-related causes” (Moberly et al., 2013) and medical complications as a possible cause for a device failure can be excluded. Radiographic examinations do not reveal any indication of device migration or electrode displacement. Finally, the problems disappear after reimplantation (Moberly et al., 2013).

6 According to the FDA, the patient will “…have to use [the device] for the rest of life.”

7 [CI: 23.5% for traditional victimization and 8.8% for cyber-victimization; hearing: 9.3 and 6.9%, respectively; Rodríguez-Hidalgo et al. (2019) and Feijóo et al. (2021)].

8 See for example the information of the American Cochlear Implant Alliance https://www.acialliance.org/page/telehealth.

9 According to the FDA the CI “…will interact with the electronic environment., be affected by cellular phone users or other radio transmitters, … interact in unpredictable ways with other computer systems.”

## References

[B1] Archbold S. M. Mark E. Lutman M. E. Gregory S. O’Neill C. Nikolopoulos T. P. (2002). Parents and their deaf child: Their perceptions three years after cochlear implantation. *Deaf. Educ. Int.* 4 12–40. 10.1179/146431502790560962

[B2] Bakas T. McLennon S. M. Carpenter J. S. Buelow J. M. Hanna K. M. (2012). Systematic review of health-related quality of life models. *Health Qual. Life Outcomes* 16:134. 10.1186/1477-7525-10-134 23158687 PMC3548743

[B3] Balkany T. J. Hodges A. V. Buchman C. A. Luxford W. M. Pillsbury C. H. Roland P. S. (2005). Cochlear Implant Soft Failures Consensus Development Conference Statement. *Otol. Neurotol.* 26 815–818. 10.1097/01.mao.0000178150.44505.52 16015190

[B4] Barker A. B. Leighton P. Ferguson M. A. (2017). Coping together with hearing loss: A qualitative meta-synthesis of the psychosocial experiences of people with hearing loss and their communication partners. *Int. J. Audiol.* 56 297–305. 10.1080/14992027.2017.1286695 28599604

[B5] Beacham B. L. Deatrick J. A. (2015). Children with chronic conditions: Perspectives on condition management. *J. Pediatr. Nurs.* 30 25–35. 10.1016/j.pedn.2014.10.011 25458105 PMC4291290

[B6] Bess F. H. Gustafson S. J. Corbett B. A. Lambert E. W. Camarata S. M. Hornsby B. W. (2016). Salivary cortisol profiles of children with hearing loss. *Ear Hear.* 37 334–344. 10.1097/AUD.0000000000000256 26684396 PMC4844856

[B7] Bhadania S. R. Vishwakarma R. Keshri A. (2018). Cochlear Implant Device Failure in the Postoperative Period: An Institutional Analysis. *Asian. J. Neurosurg*. 13 1066–1070. 10.4103/ajns.AJNS_93_17 30459869 PMC6208201

[B8] Blackwell C. K. Ganiban J. Herbstman J. Hunt K. Forrest C. B. (2019). General Health and Life Satisfaction in Children With Chronic Illness. *Pediatrics* 143:e20182988. 10.1542/peds.2018-2988 31061222 PMC6564050

[B9] Burwell S. Sample M. Racine E. (2017). Ethical aspects of brain computer interfaces: A scoping review. *BMC Med. Ethics* 18:60. 10.1186/s12910-017-0220-y 29121942 PMC5680604

[B10] Capkun S. Bodner D. (2010). *On The Security and Privacy Risks in Cochlear Implants.* Zurich: ETH, Department of Computer Science.

[B11] Cardon G. Sharma A. (2019). Somatosensory Cross-Modal Reorganization in Children With Cochlear Implants. *Front. Neurosci.* 26:469. 10.3389/fnins.2019.00469 31312115 PMC6613479

[B12] Cejas I. Coto J. Sarangoulis C. Sanchez C. M. Quittner A. L. (2021). Quality of Life-CI: Development of an Early Childhood Parent-Proxy and Adolescent Version. *Ear Hear.* 42 1072–1083. 10.1097/AUD.0000000000001004 33974778 PMC8855668

[B13] Chen J. Chen B. Shi Y. Li Y. (2022). A retrospective review of cochlear implant revision surgery: A 24-year experience in China. *Eur. Arch. Otorhinolaryngol.* 279 1211–1220. 10.1007/s00405-021-06745-1 33813626

[B14] Ching T. Y. C. Cupples L. Leigh G. Hou S. Wong A. (2021). Predicting Quality of Life and Behavior and Emotion from Functional Auditory and Pragmatic Language Abilities in 9-Year-Old Deaf and Hard-of-Hearing Children. *J. Clin. Med.* 17:5357. 10.3390/jcm10225357 34830640 PMC8623297

[B15] Clark G. M. Tong Y. C. Black R. Forster I. C. Patrick J. F. Dewhurst D. J. (1977). A multiple electrode cochlear implant. *J. Laryngol. Otol.* 91 935–945. 10.1017/s0022215100084607 591780

[B16] Davis E. Waters E. Mackinnon A. Reddihough D. Graham H. K. Mehmet-Radji O. (2006). Paediatric quality of life instruments: A review of theimpact of the conceptual framework on outcomes. *Dev. Med. Child Neurol.* 48 311–318. 16542522 10.1017/S0012162206000673

[B17] De Lourdes Levy M. Larcher V. Kurz R. (2003). Informed consent/assent in children. Statement of the Ethics Working Group of the Confederation of European Specialists in Paediatrics (CESP). *Eur. J. Pediatr.* 162 629–633. 10.1007/s00431-003-1193-z 12884032

[B18] Deep L. Purcell P. L. Gordon K. A. Papsin B. C. Roland J. T. Jr. Waltzman S. B. (2021). Cochlear Implantation in Infants: Evidence of Safety. *Trends Hear.* 25:23312165211014695. 10.1177/23312165211014695 34028328 PMC8150451

[B19] Denning T. Matsuoka Y. Kohno T. (2009). Neurosecurity: Security and privacy for neural devices. *Neurosurg. Focus* 27:E7. 10.3171/2009.4.FOCUS0985 19569895

[B20] Desoyer I. Hochmair E. (1977). Implantable eight-channel stimulator for the deaf. *Proc. Europ. Solid State Circ. Conf.* 77 87–89.

[B21] Dettman S. J. Dowell R. C. Choo D. Arnott W. Abrahams Y. Davis A. (2016). Long-term Communication Outcomes for Children Receiving Cochlear Implants Younger Than 12 Months: A Multicenter Study. *Otol. Neurotol.* 37 e82–95. 10.1097/MAO.0000000000000915 26756160

[B22] DiNino M. O’Brien G. Bierer S. M. Jahn K. N. Arenberg J. G. (2019). The Estimated Electrode-Neuron Interface in Cochlear Implant Listeners Is Different for Early-Implanted Children and Late-Implanted Adults. *J. Assoc. Res. Otolaryngol.* 20 291–303. 10.1007/s10162-019-00716-4 30911952 PMC6513958

[B23] Drotar D. (2004). Validating measures of pediatric health status, functional status, and health-related quality of life: Key methodological challenges and strategies. *Ambul. Pediatr.* 4 358–364. 10.1367/a03-101r.1 15264947

[B24] Ellert U. Ravens-Sieberer U. Erhart M. Kurth B. M. (2011). Determinants of agreement between self-reported and parent-assessed quality of life fo children in Germany-results of the German Health Interview and Examination Survey for Children and Adolescents (KiGGS). *Health Qual. Life Outcomes* 9:102. 10.1186/1477-7525-9-102 22111939 PMC3286376

[B25] Fayed N. de Camargo O. K. Kerr E. Rosenbaum P. Dubey A. Bostan C. (2012). Generic patient-reported outcomes in child health research: A review of conceptual content using World Health Organization definitions. *Dev. Med. Child Neurol.* 54 1085–1095. 10.1111/j.1469-8749.2012.04393 22913566

[B26] Feijóo S. Foody M. Pichel R. Zamora L. Rial A. (2021). Bullying and Cyberbullying among Students with Cochlear Implants. *J. Deaf. Stud. Deaf. Educ.* 1 130–141. 10.1093/deafed/enaa029 32978624

[B27] Fitzpatrick E. M. Jacques J. Neuss D. (2011). Parental perspectives on decision-making and outcomes in pediatric bilateral cochlear implantation. *Int. J. Audiol.* 50 679–687. 10.3109/14992027.2011.590823 21812634

[B28] Geers A. E. Nicholas J. Tobey E. Davidson L. (2016). Persistent language delay versus late language emergence in children with early cochlear implantation. *J. Speech Lang. Hear. Res.* 59 155–170. 10.1044/2015-JSLHRH-14-0173 26501740 PMC4867929

[B29] Hansson S. O. (2020). The Ethics of Cranial Nerve Implants. *Otolaryngol. Clin. North Am.* 53 21–30. 10.1016/j.otc.2019.09.001 31648823

[B30] Harris J. M. Neault M. W. O’Neill E. E. Griffin A. M. Kawai K. Kenna M. A. (2020). Incidence, Time Course, and Implications of Electrode Abnormalities in Pediatric Cochlear Implant Recipients. *Ear Hear.* 42 334–342. 10.1097/AUD.0000000000000924 32826503

[B31] Haukedal C. L. Lyxell B. Wie O. B. (2020). Health-Related Quality of Life With Cochlear Implants: The Children’s Perspective. *Ear Hear.* 41 330–343. 10.1097/AUD.0000000000000761 31408046

[B32] Haukedal C. L. Von Koss Torkildsen J. Lyxell B. Wie O. B. (2018). Parents’ Perception of Health-Related Quality of Life in Children With Cochlear Implants: The Impact of Language Skills and Hearing. *J. Speech Lang. Hear. Res.* 61 2084–2098. 10.1044/2018_JSLHR-H-17-0278 30046806

[B33] Hoffman M. F. Cejas I. Quittner A. L. (2019). Health-Related Quality of Life Instruments for Children With Cochlear Implants: Development of Child and Parent-Proxy Measures. *Ear Hear.* 40 592–604. 10.1097/AUD.0000000000000631 30059365 PMC6348146

[B34] Huber M. (2005). Health-related quality of life of Austrian children and adolescents with cochlear implants. *Int. J. Pediatr. Otorhinolaryngol.* 69 1089–1101. 10.1016/j.ijporl.2005.02.018 15946746

[B35] Huber M. Burger T. Illg A. Kunze S. Giourgas A. Braun L. (2015). Mental health problems in adolescents with cochlear implants: Peer problems persist after controlling for additional handicaps. *Front. Psychol.* 6:953. 10.3389/fpsyg.2015.00953 26236251 PMC4502340

[B36] Huber M. Hitzl W. Albegger K. (2008). Education and training of young people who grew up with cochlear implants. *Int. J. Pediatr. Otorhinolaryngol.* 72 1393–1403. 10.1016/j.ijporl.2008.06.002 18635271

[B37] Huber M. Kipman U. (2012). Cognitive skills and academic achievement of deaf children with cochlear implants. *Otolaryngol. Head Neck Surg.* 147 763–772. 10.1177/0194599812448352 22623402

[B38] Huber M. Kipman U. Pletzer B. (2014). Reading instead of reasoning? Predictors of arithmetic skills in children with cochlear implants. *Int. J. Pediatr. Otorhinolaryngol.* 78 1147–1152. 10.1016/j.ijporl.2014.04.038 24861020

[B39] Humes L. E. Dubno J. R. (2021). A Comparison of the Perceived Hearing Difficulties of Community and Clinical Samples of Older Adults. *J. Speech Lang. Hear. Res.* 14 3653–3667. 10.1044/2021_JSLHR-20-00728 34428100 PMC8642086

[B40] Iffland B. Sansen L. M. Catani C. Neuner F. (2014). The trauma of peer abuse: Effects of relational peer victimization and social anxiety disorder on physiological and affective reactions to social exclusion. *Front. Psychiatry* 5:26. 10.3389/fpsyt.2014.00026 24672491 PMC3957367

[B41] Kral A. Kronenberger W. G. Pisoni D. B. O’Donoghue G. M. (2016). Neurocognitive factors in sensory restoration of early deafness: A connectome model. *Lancet Neurol.* 15 610–621. 10.1016/S1474-4422(16)00034-X 26976647 PMC6260790

[B42] Kral A. Sharma A. (2012). Developmental neuroplasticity after cochlear implantation. *Trends Neurosci.* 35 111–122. 10.1016/j.tins.2011.09.004 22104561 PMC3561718

[B43] Lammers M. J. Jansen T. T. Grolman W. Lenarz T. Versnel H. Van Zanten G. A. (2015). The Influence of Newborn Hearing Screening on the Age at Cochlear Implantation in Children. *Laryngoscope* 125 985–990. 10.1002/lary.25045 25676285

[B44] Lander D. P. Durakovic N. Kallogjeri D. Jiramongkolchai P. Olsen M. A. Piccirillo J. F. (2020). Incidence of Infectious Complications Following Cochlear Implantation in Children and Adults. *JAMA* 323 182–183. 10.1001/jama.2019.18611 31935017 PMC6990693

[B45] Lane C. Zimmerman K. Agrawal S. Parnes L. (2019). Cochlear implant failures and reimplantation: A 30-year analysis and literature review. *Laryngoscope* 130 782–789. 10.1002/lary.28071 31112331

[B46] Lee H. J. Smieja D. Polonenko M. J. Cushing S. L. Papsin B. C. Gordon K. A. (2020). Consistent and chronic cochlear implant use partially reverses cortical effects of single sided deafness in children. *Sci. Rep.* 10:21526. 10.1038/s41598-020-78371-6 33298987 PMC7726152

[B47] Lieu J. E. C. Kenna M. Anne S. Davidson L. (2020). Hearing Loss in Children: A Review. *JAMA* 324 2195–2205. 10.1001/jama.2020.17647 33258894

[B48] Mai P. T. Tick A. (2021). Cyber Security Awareness and Behavior of Youth in Smartphone Usage: A Comparative Study between University Students in Hungary and Vietnam. *Acta Polytech. Hung.* 18 67–89. 10.12700/APH.18.8.2021.8.4

[B49] Moberly A. C. Welling D. B. Nittrouer S. (2013). Detecting soft failures in pediatric cochlear implants: Relating behavior to language outcomes. *Otol. Neurotol.* 34 1648–1655. 10.1097/MAO.0b013e3182a0036c 24136308 PMC3830631

[B50] Naik A. N. Varadarajan V. V. Malhotra P. S. (2021). Early pediatric Cochlear implantation: An update. *Laryngoscope Investig. Otolaryngol.* 12 512–521. 10.1002/lio2.574 34195373 PMC8223461

[B51] Nisenbaum E. J. Roland J. T. Waltzman S. Friedmann D. R. (2020). Risk Factors and Management of Postoperative Infection Following Cochlear Implantation. *Otol. Neurotol.* 41 e823–e828. 10.1097/MAO.0000000000002685 32658104

[B52] Ojelabi A. O. Graham Y. Haighton C. Ling J. (2017). A systematic reviewof the application of Wilson and Cleary health-related quality of life model in chronic diseases. *Health Qual. Life Outcomes* 11:241. 10.1186/s12955-017-0818-2 29228977 PMC5725920

[B53] Okubo S. Takahashi M. Kai I. (2008). How Japanese parents of deaf children arrive at decisions regarding pediatric cochlear implantation surgery: A qualitative study. *Soc. Sci. Med.* 66 2436–2447. 10.1016/j.socscimed.2008.02.013 18362048

[B54] Percy-Smith L. Cayé-Thomasen P. Gudman M. Jensen J. H. Thomsen J. (2008). Self-esteem and social well-being of children with cochlear implant compared to normal-hearing children. *Int. J. Pediatr. Otorhinolaryngol.* 72 1113–1120. 10.1016/j.ijporl.2008.03.028 18501436

[B55] Peters B. R. Wyss J. Manrique M. (2010). Worldwide trends in bilateral cochlear implantation. *Laryngoscope* 120 S17–S44. 20422715 10.1002/lary.20859

[B56] Pontillo M. Tata M. C. Averna R. Demaria F. Gargiullo P. Guerrera S. (2019). Peer Victimization and Onset of Social Anxiety Disorder in Children and Adolescents. *Brain Sci.* 6:132. 10.3390/brainsci9060132 31174384 PMC6627045

[B57] Purcell P. L. Deep N. L. Waltzman S. B. Roland J. T. Jr. Cushing S. L. Papsin B. C. (2021). Cochlear Implantation in Infants: Why and How. *Trends Hear.* 25:23312165211031751. 10.1177/23312165211031751 34281434 PMC8295935

[B58] Rachakonda T. Jeffe D. B. Shin J. J. Mankarious L. Fanning R. J. Lesperance M. M. (2014). Validity, discriminative ability, and reliability of the hearing-related quality of life questionnaire for adolescents. *Laryngoscope* 124 570–578. 10.1002/lary.24336 23900836 PMC3951892

[B59] Rajan G. Tavora-Vieira D. Baumgartner W. D. Godey B. Mueller J. O’Driscoll M. (2018). Hearing preservation cochlear implantation in children: The HEARRING Group consensus and practice guide. *Cochlear. Implants Int.* 19 1–13. 10.1080/14670100.2017.1379933 29073844

[B60] Ravens-Sieberer U. Devine J. Bevans K. Riley A. W. Moon J. Salsman J. M. (2014a). Subjective wellbeing measures for children were developed within the PROMIS project: Presentation of first results. *J. Clin. Epidemiol.* 67 207–218. 10.1016/j.jclinepi.2013.08.018 24295987 PMC4120943

[B61] Ravens-Sieberer U. Karow A. Barthel D. Klasen F. (2014b). How to assess quality of life in child and adolescent psychiatry. *Dialogues Clin. Neurosci.* 16 147–158. 10.31887/DCNS.2014.16.2/usieberer 25152654 PMC4140509

[B62] Rebok G. Riley A. Forrest C. Starfield B. Green B. Robertson J. (2001). Elementary school-aged children’s reports of their health: A cognitiveinterviewing study. *Qual. Life Res.* 10 59–70. 10.1023/a:1016693417166 11508476

[B63] Riley A. W. (2004). Evidence that school-age children can self-report on their health. *Ambul. Pediatr.* 4 371–376. 10.1367/a03-178r.1 15264962

[B64] Rodríguez-Hidalgo A. J. Calmaestra J. Casas J. A. Ortega-Ruiz R. (2019). Ethnic-cultural bullying versus personal bullying: Specificity and measurement of discriminatory aggression and victimization among adolescents. *Front. Psychol.* 10:46. 10.3389/fpsyg.2019.00046 30774605 PMC6367499

[B65] Ruben R. J. (2018). Language development in the pediatric cochlear implant patient. *Laryngoscope Investig. Otolaryngol.* 19 209–213. 10.1002/lio2.156 30062136 PMC6057214

[B66] Sarant J. Z. Harris D. C. Bennet L. A. (2015). Academic outcomes for school-aged children with severe-profound hearing loss and early unilateral and bilateral cochlear implants. *J. Speech Lang. Hear. Res.* 58 1017–1032. 10.1044/2015_JSLHR-H-14-0075 25677804

[B67] Shader M. J. Gordon-Salant S. Goupell M. J. (2020). Impact of Aging and the Electrode-to-Neural Interface on Temporal Processing Ability in Cochlear-Implant Users: Gap Detection Thresholds. *Trends Hear.* 24:233121652095656. 10.1177/2331216520956560 32941111 PMC7502859

[B68] Sharma A. Dorman M. F. Spahr A. J. (2002). A sensitive period for the development of the central auditory system in children with cochlear implants: Implications for age of implantation. *Ear Hear.* 23 532–539. 10.1097/00003446-200212000-00004 12476090

[B69] Sharma S. D. Cushing S. L. Papsin B. C. Gordon K. A. (2020). Hearing and speech benefits of cochlear implantation in children: A review of the literature. *Int. J. Pediatr. Otorhinolaryngol*. 133:109984. 10.1016/j.ijporl.2020.109984 32203759

[B70] Suneel D. Davidson L. S. Lieu J. (2020). Self-reported hearing quality of life measures in pediatric cochlear implant recipients with bilateral input. *Cochlear. Implants Int.* 21 83–91. 10.1080/14670100.2019.1670486 31590628 PMC7002198

[B71] Tabasum A. Safi Z. AIKhater W. Shikfa A. (2018). “Cybersecurity Issues in Implanted Medical Devices,” in *International Conference on Computer and Applications (ICCA).* (New York, NY: IEEE), 1–9. 10.1109/COMAPP.2018.8460454

[B72] Theunissen S. C. Rieffe C. Kouwenberg M. De Raeve L. Soede W. Briaire J. J. (2012). Anxiety in children with hearing aids or cochlear implants compared to normally hearing controls. *Laryngoscope* 122 654–659. 10.1002/lary.22502 22252674

[B73] Uecker F. C. Szczepek A. Olze H. (2019). Pediatric Bilateral Cochlear Implantation: Simultaneous Versus Sequential Surgery. *Otol. Neurotol.* 40 e454–e460. 10.1097/MAO.0000000000002177 30870380

[B74] Umansky A. M. Jeffe D. B. Lieu J. E. (2011). The HEAR-QL: Quality of life questionnaire for children with hearing loss. *J. Am. Acad. Audiol.* 22 644–653. 10.3766/jaaa.22.10.3 22212764 PMC3273903

[B75] Upton P. Lawford J. Eiser C. (2008). Parent-child agreement across child health-related quality of life instruments: A review of the literature. *Qual. Life Res.* 17 895–913. 10.1007/s11136-008-9350-5 18521721

[B76] Van der Straaten T. F. K. Rieffe C. Soede W. Netten A. P. Dirks E. Oudesluys-Murphy A. M. (2020). Quality of life of children with hearing loss in special and mainstream education: A longitudinal study. *Int. J. Pediatr. Otorhinolaryngol.* 128:109701. 10.1016/j.ijporl.2019.109701 31606686

[B77] Vas V. Akeroyd M. A. Hall D. A. (2017). A Data-Driven Synthesis of Research Evidence for Domains of Hearing Loss, as Reported by Adults With Hearing Loss and Their Communication Partners. *Trends Hear.* 21:2331216517734088. 10.1177/2331216517734088 28982021 PMC5638151

[B78] Vila P. M. Ghogomu N. T. Odom-John A. R. Hullar T. E. Hirose K. (2017). Infectious complications of pediatric cochlear implants are highly influenced by otitis media. *Int. J. Pediatr. Otorhinolaryngol.* 97 76–82. 10.1016/j.ijporl.2017.02.026 28483256 PMC6198317

[B79] Wallander J. L. Koot H. M. (2016). Quality of life in children: A critical examination of concepts, approaches, issues, and future directions. *Clin. Psychol. Rev.* 45 131–143. 10.1016/j.cpr.2015.11.007 26911191

[B80] Wang Y. Liu L. Zhang Y. Wei C. Xin T. He Q. (2021). The Neural Processing of Vocal Emotion After Hearing Reconstruction in Prelingual Deaf Children: A Functional Near-Infrared Spectroscopy Brain Imaging Study. *Front. Neurosci.* 28:705741. 10.3389/fnins.2021.705741 34393716 PMC8355545

[B81] Warner-Czyz A. D. Loy B. Pourchot H. White T. Cokely E. (2018). Effect of hearing loss on peer victimization in school-age children. *Except. Child* 84 280–297. 10.1177/0014402918754880

[B82] Wheeler A. Archbold S. Gregory S. Skipp A. (2007). Cochlear implants: The young people’s perspective. *J. Deaf. Stud. Deaf. Educ.* 12 303–316. 10.1093/deafed/enm018 17533173

[B83] Wiebe S. Guyatt G. Weaver B. Matijevic S. Sidwell C. J. (2003). Comparative responsiveness of generic and specific quality-of-life instruments. *Clin. Epidemiol.* 56 52–60. 10.1016/s0895-4356(02)00537-1 12589870

[B84] Wilson I. B. Cleary P. D. (1995). Linking clinical variables with health-relatedquality of life. A conceptual model of patient outcomes. *JAMA* 273 59–65. 10.1001/jama.273.1.59 7996652

[B85] Wollenhaupt J. Rodgers B. Sawin K. J. (2012). Family management of a chronic health condition: Perspectives of adolescents. *J. Fam. Nurs.* 18 65–90. 10.1177/1074840711427545 22184753

[B86] World Health Organization [Who] (1948). *WHO Definition of Health, Preamble to the Constitution of the World Health Organization as Adopted by the International Health Conference.* Geneva: World Health Organization.

[B87] World Health Organization [Who] (1994). *Measurement of Quality of Life inChildren. MNH/PSF/94.5.* Geneva: World Health Organization.

[B88] Yeung J. Griffin A. Newton S. Kenna M. Licameli G. R. (2018). Revision cochlear implant surgery in children: Surgical and audiological outcomes. *Laryngoscope* 128 2619–2624. 10.1002/lary.27198 29729014

[B89] Yosefof E. Hilly O. Ulanovski D. Raveh E. Attias J. Sokolov M. (2021). Cochlear implant failure: Diagnosis and treatment of soft failures. *Acta Otorhinolaryngol. Ital.* 41 566–571. 10.14639/0392-100X-N1583 34928268 PMC8686795

